# Incidence and Risk Factors of Diabetic Retinopathy in Patients with Type 1 Diabetes Mellitus: A Retrospective Study in NGHA, Riyadh, Saudi Arabia

**DOI:** 10.3390/jcm15103811

**Published:** 2026-05-15

**Authors:** Inam Ul-Haq, Hassan S. Alqahtani, Naila A. Shaheen, Meshal S. Alghamdi, Sultan A. Aldosari, Abdulrahman S. Altowaim, Naif H. Alqadhy, Abdulaziz M. Alqahtani, Mohammed Bukhaytan, Muhammad Imran Khan, Maliha Rani

**Affiliations:** 1 Department of Ophthalmology, Ministry of National Guard-Health Affairs, Riyadh 11426, Saudi Arabia; 2 College of Medicine, King Saud bin Abdulaziz University for Health Sciences, Riyadh 11481, Saudi Arabia421110231@ksau-hs.edu.sa (A.M.A.);; 3 Division of Biostatistics, Department of Population Health, King Abdullah International Medical Research Center, King Saud Bin Abdulaziz University for Health Sciences (KSAU-HS), Ministry of National Guard Health Affairs (MNGHA), Riyadh 11426, Saudi Arabia

**Keywords:** type 1 diabetes mellitus, diabetic retinopathy, risk factors, glycemic variability, Saudi Arabia

## Abstract

**Background/Objectives:** Diabetic retinopathy (DR) is a major microvascular complication of type 1 diabetes mellitus (T1DM) and remains an important cause of preventable visual impairment. Region-specific data on the incidence and clinical predictors of DR among patients with T1DM in Saudi Arabia remain limited. This study aimed to determine the incidence of DR and identify associated demographic and systemic risk factors among patients with T1DM at a tertiary care center in Riyadh, Saudi Arabia. **Methods:** This retrospective cohort study included 449 patients with T1DM aged ≥9 years who were followed at King Abdulaziz Medical City, Riyadh, between 2015 and 2025. Patients were selected using a consecutive non-probability sampling technique. Data were extracted from the BESTCare 2.0A electronic medical record system and supplemented, when required, by phone-based interviews to verify selected clinical and demographic variables. Patients were classified as controls without DR or cases with DR, including non-vision-threatening DR and vision-threatening DR (VTDR), according to the International Clinical Diabetic Retinopathy Severity Scale. Multivariable logistic regression, Cox proportional hazards models, and temporal trend analysis were performed, with statistical significance set at *p* < 0.05. **Results:** The overall incidence rate of DR was 92.66 per 1000 person-years, with similar rates among males and females. In multivariable logistic regression, older age at T1DM diagnosis, longer diabetes duration, hypertension, hyperlipidemia, and albuminuria were independently associated with DR. Mean HbA1c and HbA1c variability were not independently associated with DR after adjustment. In Cox regression, older age at T1DM diagnosis was associated with higher hazards of both DR and VTDR, while hypertension was associated with VTDR. Among patients with DR, younger age at T1DM diagnosis was associated with higher odds of proliferative disease in exploratory severity analysis. **Conclusions:** DR was common among patients with T1DM in this tertiary-care cohort and was mainly associated with disease duration, age at diagnosis, and systemic vascular comorbidities. These findings support the importance of routine ophthalmologic screening and integrated management of systemic risk factors in patients with T1DM.

## 1. Introduction

Type 1 diabetes mellitus (T1DM) is a chronic autoimmune disease characterized by the destruction of pancreatic β-cells, resulting in absolute insulin deficiency and lifelong dependence on exogenous insulin therapy [1]. Despite advances in diabetes management, long-term complications remain a major cause of morbidity. Among these, diabetic retinopathy (DR) represents one of the most serious microvascular complications and is a leading cause of visual impairment and blindness in individuals with diabetes. Visual loss related to DR may result from macular edema, vitreous hemorrhage, retinal detachment, and neovascular glaucoma [2,3,4,5]. The risk of developing DR increases significantly with longer disease duration and poor metabolic control [6].

Globally, diabetic retinopathy is one of the principal causes of vision loss among adults and continues to pose a substantial public health burden [2,3,4]. In addition, as the incidence of diabetes, including T1DM, continues to rise rapidly, Saudi Arabia is expected to face further healthcare challenges. Given that Saudi Arabia has one of the highest diabetes rates worldwide, diabetes-related complications, particularly diabetic retinopathy, require urgent attention [7].

Diabetic retinopathy (DR) is a complex multifactorial complication influenced by several systemic and ocular risk factors. A recent Cochrane review evaluated prognostic factors for the development and progression of proliferative diabetic retinopathy (PDR) among individuals with pre-existing DR. The review found that increased glycated hemoglobin (HbA1c) levels and more advanced baseline DR severity were likely associated with a higher risk of progression to PDR in patients with T1DM and type 2 diabetes mellitus (T2DM). Renal impairment in individuals with T1DM or T2DM, as well as younger age at diabetes diagnosis, increased triglyceride levels, and larger retinal venular diameters among individuals with T1DM, may also be associated with an increased risk of progression to PDR [8]. These findings support the importance of early DR detection, adequate glycemic control, and management of systemic risk factors to reduce the risk of sight-threatening disease.

Similarly, recent T1DM-specific evidence has emphasized the importance of diabetes duration, poor glycemic control, younger age at diabetes onset, and irregular ophthalmologic examinations in the development of proliferative diabetic retinopathy, supporting the need for structured screening and risk stratification in this population [9]. Moreover, a local study conducted at a diabetes healthcare facility in Saudi Arabia included 502 patients with diabetes mellitus, of whom 174 had T1DM. Logistic regression analysis identified nephropathy, insulin use, longer diabetes duration (>10 years), poor glycemic control, and older age (>60 years) as major risk factors for DR. Long-standing diabetes was the strongest independent predictor of retinopathy development, while nephropathy was strongly associated with disease severity [10]. More recently, a Saudi systematic review and meta-analysis reported a substantial burden of DR in Saudi Arabia and identified longer diabetes duration, poor glycemic control, obesity, and hypertension as important predictors, further highlighting the need for local evidence to guide screening and prevention strategies [11]. Overall, these findings emphasize the close relationship between microvascular complications and systemic disease burden in patients with diabetes.

In parallel, recent advances in retinal imaging have expanded opportunities for earlier detection and image-based classification of diabetic retinopathy. Wang et al. demonstrated the potential role of hyperspectral imaging combined with image-processing and principal component analysis techniques in identifying diabetic retinopathy-related retinal vascular changes and distinguishing disease stages [12]. However, these imaging-based approaches still require integration with clinically relevant and locally validated risk profiles to identify patients who may benefit from intensified surveillance.

Despite the growing body of international and regional research, the available evidence remains partly limited by differences in population characteristics, diabetes type, study design, screening methods, and healthcare settings. Much of the international evidence is derived from non-Saudi populations, mixed diabetes cohorts, or screening-based datasets, which may differ from local patients with T1DM in demographic structure, age at disease onset, systemic comorbidity burden, referral patterns, and access to ophthalmic care. In Saudi Arabia, available studies have mainly reported diabetic retinopathy prevalence and associated risk factors in mixed diabetes populations or type 2 diabetes mellitus-focused cohorts. However, local ophthalmology-focused data specifically evaluating DR incidence, vision-threatening diabetic retinopathy, proliferative diabetic retinopathy severity patterns, and independent clinical predictors among patients with T1DM remain limited.

Region-specific data are therefore essential to better understand disease patterns, support risk stratification, and optimize preventive strategies. Accordingly, the present study aims to determine the incidence of diabetic retinopathy among patients with T1DM and to identify associated demographic and clinical risk factors in a tertiary care center in Riyadh, Saudi Arabia. In addition, this study evaluates clinical and demographic characteristics and their association with the development and progression of DR. By providing locally relevant evidence, this research aims to support early detection strategies, improve patient management, and inform healthcare planning to reduce the burden of diabetic retinopathy in Saudi Arabia.

## 2. Materials and Methods

### 2.1. Study Design and Setting

The current study employed a retrospective cohort design to evaluate risk factors associated with the development and progression of diabetic retinopathy (DR) among patients with type 1 diabetes mellitus (T1DM). This study was conducted at the Department of Ophthalmology, King Abdulaziz Medical City (KAMC), Riyadh, Saudi Arabia, a leading tertiary care center serving National Guard Health Affairs (NGHA) beneficiaries. Data were systematically extracted from BESTCare 2.0A, the hospital’s integrated electronic medical record system, including relevant clinical and ophthalmic records. This study included patients who were followed between 2015 and 2025.

### 2.2. Participants and Selection Criteria

The study population consisted of patients diagnosed with T1DM who received care at KAMC. Using a consecutive non-probability sampling method, participants were included if they were aged 9 years or older, had a confirmed diagnosis of T1DM, and had complete electronic medical records, including at least one documented ophthalmic examination. All included participants had T1DM; therefore, the control group did not consist of healthy individuals. Controls were defined as patients with T1DM who had no documented evidence of diabetic retinopathy, including diabetic macular edema, on ophthalmic examination. Cases were defined as patients with T1DM who had documented diabetic retinopathy, including diabetic macular edema. Patients were excluded if their medical records were incomplete or lacked critical laboratory data, such as HbA1c levels.

### 2.3. Ethical Approval

This study was approved by the Institutional Review Board of King Abdullah International Medical Research Center (KAIMRC), Ministry of National Guard Health Affairs, Riyadh, Saudi Arabia (protocol code: NRC23R/861/12; approval date: 26 March 2024). This study was conducted in accordance with the Declaration of Helsinki and applicable institutional and national research ethics regulations. The period from 2015 to 2025 refers to the historical timeframe of the clinical records reviewed. All active research procedures, including BESTCare 2.0A data extraction and structured telephone confirmation of selected variables, such as smoking status and family history, were conducted only after IRB approval. The requirement for written informed consent was waived due to the retrospective nature of the study; however, verbal informed consent was obtained from patients before proceeding with the telephone interview. The telephone-based data collection procedures were conducted as part of the IRB-approved study protocol.

### 2.4. Variables and Measurement

The primary outcome of this study was the development and progression of diabetic retinopathy (DR). All participants underwent complete, standardized ophthalmic examinations at least once per year, with more frequent evaluations for those at high risk of visual decline.

The stages of DR and diabetic macular edema (DME) were determined according to the International Clinical Diabetic Retinopathy Severity Scale [13]. Participants were categorized into three analytical groups: controls with no DR, non-vision-threatening diabetic retinopathy (NVTDR), and vision-threatening diabetic retinopathy (VTDR). NVTDR was defined as mild or moderate non-proliferative DR (NPDR) or DME distant from the fovea. VTDR was defined by the presence of severe NPDR, corresponding to the 4-2-1 rule, proliferative DR (PDR), or DME with hard exudates approaching or involving the fovea [13].

Systemic clinical variables and comorbidities were identified using standardized diagnostic cut-offs and verified medical records within the BESTCare 2.0A system. Hypertension was defined as a recorded systolic blood pressure ≥ 130 mmHg or diastolic blood pressure ≥ 80 mmHg on two or more separate clinical visits, concurrent use of antihypertensive medications, or a documented diagnosis in the system notes [14]. Hyperlipidemia was defined using ATP III lipid classification thresholds as total cholesterol > 5.2 mmol/L (>200 mg/dL), LDL cholesterol > 3.4 mmol/L (>130 mg/dL), triglycerides ≥ 1.7 mmol/L (≥150 mg/dL), or a documented diagnosis requiring lipid-lowering therapy [15]. Albuminuria was defined as a persistent albumin-to-creatinine ratio (ACR) ≥ 30 mg/g, a 24 h urine albumin level ≥ 30 mg, or a documented diagnosis of diabetic nephropathy [16]. Body mass index (BMI) was calculated as weight (kg)/height (m^2^), with obesity defined as BMI ≥ 30.0 kg/m^2^ [17].

Glycemic status was evaluated using the mean of the latest five consecutive available glycated hemoglobin (HbA1c) readings for controls and five consecutive available HbA1c readings obtained within approximately one year around the date of DR diagnosis for cases, rather than exclusively after diagnosis. Glycemic variability was quantified using the standard deviation (SD) and coefficient of variation (CV) of these readings [18].

Smoking status was categorized based on self-reported active use at the time of assessment or documented status in the patient’s medical record. Family history of diabetes was defined as a confirmed diagnosis of type 1 diabetes, type 2 diabetes, or both in first-degree relatives, as reported by the patient during the telephone interview or noted in the clinical history.

### 2.5. Bias and Study Size

The sample size was initially calculated using a standard sample size formula based on a 95% confidence level, a 5% margin of error, and an expected proportion of 50%, yielding a minimum required sample size of 377 participants. The final analysis included a larger cohort of 449 patients, comprising 268 controls and 181 cases, which improved the precision of the estimates and allowed comparison of risk factors between groups.

Several measures were taken to minimize potential sources of bias and support the internal validity of the findings. Selection bias was reduced by using consecutive non-probability sampling and applying predefined inclusion and exclusion criteria based on data available in the BESTCare 2.0A electronic medical record system. To reduce recall bias for subjective variables, such as smoking status and family history, a structured telephone interview protocol was used, and patient responses were cross-verified with existing medical record documentation whenever available. Information bias was minimized by using objective, laboratory-verified data for clinical variables and by calculating the means of several HbA1c readings to reduce the influence of short-term fluctuations. Finally, patients with incomplete ophthalmic examinations or missing core laboratory data were excluded from the final cohort to maintain data completeness and integrity.

### 2.6. Statistical Analysis

Statistical analysis was performed using the Statistical Package for the Social Sciences (SPSS), version 23.0 (IBM Corp., Armonk, NY, USA). The normality of continuous variables was assessed using the Shapiro–Wilk test. Normally distributed continuous variables were presented as mean ± standard deviation and compared using one-way analysis of variance (ANOVA), whereas non-normally distributed variables were presented as median and interquartile range (IQR) and compared using the Kruskal–Wallis test. Categorical variables were expressed as frequencies and percentages, and group differences were evaluated using the chi-square test or Fisher’s exact test, as appropriate.

To identify independent risk factors, multivariable logistic regression models were constructed. The primary logistic regression model evaluated factors independently associated with diabetic retinopathy by comparing cases with controls. A secondary logistic regression model evaluated factors associated with vision-threatening diabetic retinopathy compared with non-vision-threatening diabetic retinopathy among cases. Cox proportional hazards models were used as complementary time-to-event analyses to estimate the hazard of developing diabetic retinopathy and vision-threatening diabetic retinopathy from the time of T1DM diagnosis to the first documented ophthalmic outcome, or to the most recent eye examination for patients who did not develop the outcome. Because diabetes duration formed part of the underlying time-to-event structure, it was not included as an independent covariate in the Cox models.

To reduce redundancy in the main manuscript, additional logistic regression models using categorized clinical variables, including glycated hemoglobin and body mass index categories, are presented in Appendix A, Table A1. The proliferative diabetic retinopathy versus non-proliferative diabetic retinopathy model was retained in the main manuscript as an exploratory severity analysis among patients with diabetic retinopathy. Complete-case analysis was applied separately for each analysis; therefore, denominators may differ between baseline characteristics, regression models, incidence analyses, and graphical summaries depending on the availability of the required variables. Incidence rates were calculated based on person-years of follow-up, with confidence intervals estimated assuming a Poisson distribution.

Temporal trend analysis was conducted across three sequential available ophthalmic assessment points. Because not all patients had complete data at each assessment, this analysis was interpreted as an exploratory evaluation of the observed distribution of diabetic retinopathy stages among available records, rather than as a fixed-cohort longitudinal progression analysis. The Cochran–Armitage trend test was applied to assess ordered temporal changes across assessment points, with results interpreted in light of the varying numbers of available staged assessments at each time point. All statistical tests were two-sided, and a *p*-value < 0.05 was considered statistically significant.

## 3. Results

### 3.1. Baseline Characteristics According to Diabetic Retinopathy Classification

Table 1 summarizes the baseline demographic, metabolic, and clinical characteristics of participants according to diabetic retinopathy classification. Controls were younger (mean age, 26.7 years) and had lower BMIs (25.0 kg/m^2^) than patients with non-vision-threatening DR (NVTDR; 55.4 years; BMI, 32.1 kg/m^2^) and vision-threatening DR (VTDR; 52.4 years; BMI, 30.3 kg/m^2^), with significant differences across groups (*p* < 0.0001). Age at T1DM diagnosis was significantly higher in the NVTDR (41.0 years) and VTDR groups (36.7 years) compared with controls (11.2 years; *p* < 0.0001). Diabetes duration did not differ significantly across groups (*p* = 0.0513). Mean HbA1c levels were comparable across groups (*p* = 0.4129); however, HbA1c variability was significantly greater in the VTDR group (SD, 1.21; CV, 12.6%) compared with controls and the NVTDR group (*p* = 0.0189 and *p* = 0.0246, respectively), indicating greater glycemic variability. Total cholesterol levels differed across groups, while HDL cholesterol was significantly lower in the NVTDR (1.19 mmol/L) and VTDR groups (1.13 mmol/L) compared with controls (1.35 mmol/L; *p* < 0.0001). Family history of diabetes was more common in the retinopathy groups (*p* = 0.0004), driven primarily by family history of type 2 diabetes (*p* = 0.0377), whereas family history of type 1 diabetes alone was not significant. The prevalence of hypertension, albuminuria, and hyperlipidemia increased markedly from controls to VTDR (all *p* < 0.0001). Smoking prevalence was modest but significantly higher in the retinopathy groups (*p* = 0.0432).

### 3.2. Incidence of Diabetic Retinopathy

Table 2 shows the incidence rate of diabetic retinopathy in the cohort. The overall incidence rate of DR was 92.66 per 1000 person-years (95% CI, 79.03–106.83). Incidence rates were similar among males (93.87 per 1000 person-years; 95% CI, 73.57–115.43) and females (91.75 per 1000 person-years; 95% CI, 73.59–110.86).

### 3.3. Primary and Secondary Multivariable Logistic Regression Models for Diabetic Retinopathy and Vision-Threatening Diabetic Retinopathy

Table 3 presents the primary and secondary multivariable logistic regression analyses. The primary model (DR model) evaluated factors independently associated with diabetic retinopathy by comparing cases with controls, while the secondary model (VTDR model) evaluated factors associated with vision-threatening diabetic retinopathy by comparing VTDR with non-vision-threatening DR among patients with diabetic retinopathy. In the adjusted model for DR (Model A; *n* = 443), older age at T1DM diagnosis (OR, 1.08 per year; 95% CI, 1.05–1.11; *p* < 0.0001), longer diabetes duration (OR, 1.08 per year; 95% CI, 1.03–1.12; *p* = 0.0007), hypertension (OR, 3.73; *p* = 0.0010), hyperlipidemia (OR, 2.38; *p* = 0.0081), and albuminuria (OR, 3.79; *p* = 0.0003) were independently associated with DR. Mean HbA1c, HbA1c variability, smoking, and obesity were not independently associated with DR. In the VTDR model (Model B; *n* = 179), no evaluated variable demonstrated a statistically significant association.

### 3.4. Complementary Cox Proportional Hazards Models for Time to Diabetic Retinopathy and Vision-Threatening Diabetic Retinopathy

Table 4 summarizes complementary Cox proportional hazards models. Unlike the logistic regression models, which assessed the odds of diabetic retinopathy status, the Cox models evaluated time-to-event associations from type 1 diabetes mellitus diagnosis to the development of diabetic retinopathy or vision-threatening diabetic retinopathy. Older age at T1DM diagnosis was associated with higher hazards of both DR (HR, 1.03; *p* = 0.0001) and VTDR (HR, 1.02; *p* = 0.0328). Hypertension was significantly associated with a higher hazard of developing VTDR (HR, 1.66; *p* = 0.0485), but it was not significantly associated with the overall development of DR. No significant associations were observed for glycemic measures, hyperlipidemia, albuminuria, smoking, or obesity.

### 3.5. Exploratory Severity Analysis for Proliferative Diabetic Retinopathy

Table 5 presents an exploratory multivariable logistic regression analysis evaluating factors associated with proliferative diabetic retinopathy compared with non-proliferative diabetic retinopathy among patients with diabetic retinopathy. This analysis was performed to assess severity-related associations beyond the primary diabetic retinopathy and vision-threatening diabetic retinopathy models. Younger age at type 1 diabetes mellitus diagnosis was significantly associated with higher odds of proliferative diabetic retinopathy (OR, 0.96 per year; 95% CI, 0.94–0.99; *p* = 0.006). No other evaluated variables, including diabetes duration, HbA1c categories, hypertension, hyperlipidemia, albuminuria, smoking, or body mass index categories, showed statistically significant associations with proliferative diabetic retinopathy after adjustment. Additional categorized-variable models for diabetic retinopathy and vision-threatening diabetic retinopathy are provided in Appendix A, Table A1.

### 3.6. Temporal Trend in Diabetic Retinopathy Across Three Assessment Time Points

**Figure 1:**
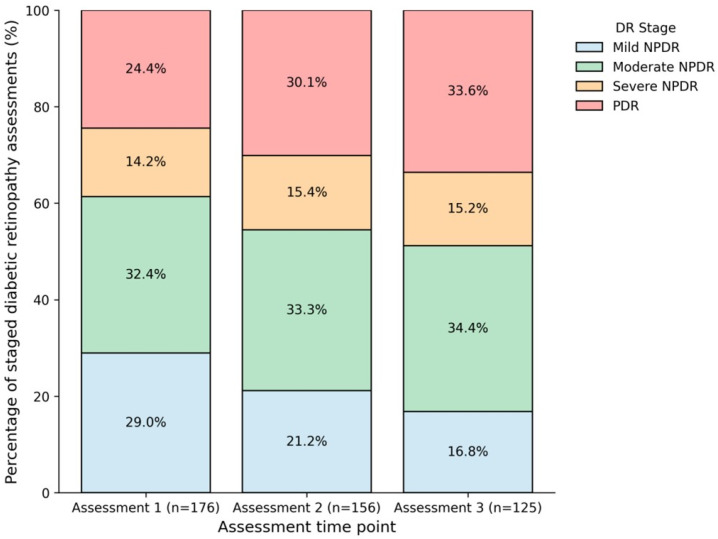
Distribution of diabetic retinopathy severity stages across sequential available ophthalmic assessments. Assessment 1, Assessment 2, and Assessment 3 represent the first, second, and third available documented ophthalmic assessments during follow-up, respectively, rather than predefined follow-up intervals. Percentages were calculated using the total number of available staged assessments at each assessment point. Because the number of patients assessed differed across assessment points, the figure reflects the observed severity distribution among available records rather than true longitudinal change within the same individuals.

Table 6 presents the distribution of diabetic retinopathy stages across three sequential available ophthalmic assessments. Assessment 1, Assessment 2, and Assessment 3 represent the first, second, and third available documented ophthalmic assessments during follow-up, respectively, rather than predefined follow-up intervals. Because the number of patients with available examinations differed across assessment points, the findings should be interpreted as changes in the observed distribution of diabetic retinopathy stages among available records rather than definitive within-patient improvement across a fixed cohort. The Cochran–Armitage trend test showed a statistically significant temporal trend (Z = −2.31, *p* = 0.021); however, this finding should be interpreted cautiously because incomplete follow-up and differences in the number of available staged assessments may have influenced the observed pattern. The corresponding percentage-based distribution of diabetic retinopathy severity stages across the sequential available ophthalmic assessments is shown in Figure 1.

### 3.7. Graphical Presentation of the Cumulative Risk of Diabetic Retinopathy and Glycemic Control Status

**Figure 2:**
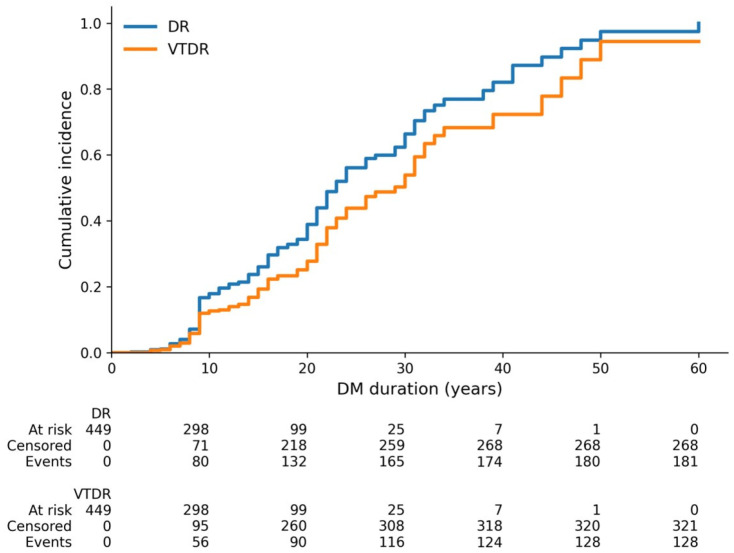
Kaplan–Meier cumulative incidence curves showing the lifetime risk of diabetic retinopathy (DR) and vision-threatening diabetic retinopathy (VTDR) according to diabetes duration. Number-at-risk tables are displayed below the curves. Abbreviations: DM, diabetes mellitus; DR, diabetic retinopathy; VTDR, vision-threatening diabetic retinopathy.

**Figure 3:**
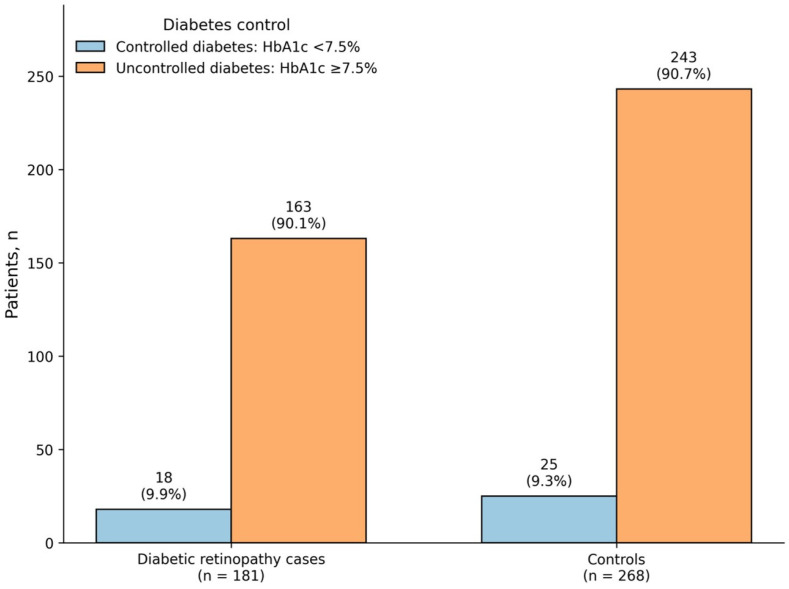
Distribution of diabetes control status among cases and controls. The figure shows the number of patients classified into each category within the case and control groups, with uncontrolled DM predominating in both groups.

Figure 2 shows that the cumulative lifetime risk of both diabetic retinopathy (DR) and vision-threatening diabetic retinopathy (VTDR) increased with a longer duration of type 1 diabetes mellitus, with DR consistently remaining more common than VTDR throughout follow-up. Figure 3 shows that uncontrolled glycemic status predominated among both cases and controls, whereas only a small proportion of patients in either group had controlled glycemic status.

## 4. Discussion

The incidence and risk factors of diabetic retinopathy (DR) among patients with type 1 diabetes mellitus (T1DM) in a tertiary care facility in Riyadh, Saudi Arabia, provide important region-specific data. The incidence rate of DR was 92.66 per 1000 person-years, with similar rates observed among males and females, suggesting that gender may not have an independent impact on the development of DR in this population. This finding is consistent with previous studies showing that gender differences in DR risk are often minimal after adjustment for metabolic and systemic factors [8,19].

One of the key findings of this study was the significant association between older age at T1DM diagnosis and the development of DR. In the multivariable logistic regression model, each one-year increase in age at T1DM diagnosis was independently associated with higher odds of DR (OR = 1.08, 95% CI 1.05–1.11, *p* < 0.0001), and this association was further supported by the Cox proportional hazards models for both DR and VTDR. This finding is consistent with Schreur et al., who reported that older age at onset of T1DM was independently associated with faster development of DR. They suggested that this association may be partly explained by age-related retinal vulnerability, whereby physiological aging of the retina may contribute to microvascular damage independently of hyperglycemia. Such age-related changes may include increased retinal vascular leakage, reduced retinal pigment epithelial integrity, and inflammatory changes within the aging retina, potentially making patients diagnosed at an older age more susceptible to diabetic retinal complications [20].

However, this finding should be distinguished from severity-related analyses. In our exploratory multivariable logistic regression analysis among patients who had already developed DR, younger age at T1DM diagnosis was significantly associated with higher odds of PDR compared with NPDR (OR = 0.96 per year, 95% CI 0.94–0.99, *p* = 0.006). This suggests that while older age at diagnosis may be related to the development of any DR in the overall cohort, younger age at diagnosis may be associated with a more advanced proliferative type among affected patients. This distinction is broadly consistent with Hietala et al., who focused specifically on the long-term risk of proliferative retinopathy and found that age at onset significantly modified the risk of PDR, with the highest risk observed among patients diagnosed between 5 and 14 years of age and the lowest risk among those diagnosed between 15 and 40 years [6]. Therefore, these findings should not be viewed as contradictory, as age at T1DM diagnosis may have different implications depending on whether the outcome is the development of any DR or the presence of advanced proliferative disease. Given the exploratory nature of the PDR analysis, this severity-related association should be interpreted cautiously and considered hypothesis-generating rather than evidence of an independent causal relationship.

The control group was younger than the case group (DR group), which likely reflects the natural history of diabetic retinopathy in T1DM, where retinal complications become more frequent with increasing age and cumulative disease exposure. This age imbalance was not due to intentional selection of younger controls but resulted from the real-world distribution of patients without DR in the retrospective cohort. Because age may influence glycemic, lipid, and systemic risk profiles, age-related and disease duration variables, including age at T1DM diagnosis and diabetes duration, were incorporated into the multivariable models. Nevertheless, residual confounding related to age cannot be fully excluded and should be considered when interpreting the findings.

Another major predictor of DR in our study was diabetes duration. Given the cumulative exposure of the retinal microvasculature to chronic hyperglycemia, logistic regression showed a higher probability of DR with longer diabetes duration, which is biologically plausible. This finding is supported by previous worldwide and regional studies that consistently identified disease duration as one of the main predictors of DR progression [6,8,10,21].

Hypertension, hyperlipidemia, and albuminuria were identified as significant independent predictors of DR among systemic comorbidities. The strongest association was observed with albuminuria (OR = 3.79), while patients with hypertension had an approximately 3.7-fold higher risk of developing DR. These results highlight the close relationship between systemic vascular dysfunction and retinal microvascular damage. Previous research has repeatedly shown that hypertension and nephropathy significantly increase the likelihood and severity of retinopathy [8,10,22].

Given that both conditions reflect widespread diabetes-related microvascular endothelial damage, the association between albuminuria and DR is biologically plausible and clinically meaningful. A strong parallel progression of diabetic nephropathy and retinopathy has been shown in previous studies [8,10]. Our findings support this relationship and suggest that albuminuria may serve as a useful clinical marker for identifying patients at high risk for retinal complications.

Interestingly, mean HbA1c was not independently associated with DR after multivariable adjustment, despite being a well-established risk factor in landmark studies [6,8,21]. Several factors may explain this finding. First, HbA1c levels were poor in both cases and controls, which may have restricted the range of values and reduced the ability to detect an independent effect. Second, the inclusion of common systemic comorbidities, such as hypertension, hyperlipidemia, and albuminuria, may have attenuated the independent association between mean HbA1c and DR in the adjusted analyses. Third, although HbA1c was summarized using five consecutive available readings, these values may not fully capture cumulative lifetime glycemic exposure, the long-term effects of earlier glycemic exposure, or glycemic fluctuations throughout the entire duration of diabetes. Therefore, the absence of an independent association between mean HbA1c and DR in this study should not be interpreted as evidence that glycemic control is not important. However, HbA1c variability was significantly higher in patients with VTDR at baseline, suggesting a possible association between glycemic instability and more severe retinopathy, although this relationship was not maintained after adjustment. This supports previous evidence suggesting that glycemic fluctuations may contribute to oxidative stress and endothelial injury beyond average glucose burden alone [18,23].

The high frequency of obesity and dyslipidemia among patients with DR is another noteworthy observation. Although obesity was not statistically significant after adjustment, its significantly higher frequency among cases raises the possibility that related metabolic syndrome pathways may contribute to disease development. Nevertheless, the role of obesity as an independent risk factor for diabetic retinopathy in T1DM remains uncertain, with prior studies showing conflicting findings [8,24].

The temporal trend analysis showed a statistically significant change in the observed distribution of diabetic retinopathy stages across the three sequential available ophthalmic assessments. However, this finding should be interpreted cautiously because the number of patients assessed at each time point was not identical, and the intervals between assessments may have varied. Therefore, the observed pattern may reflect changes in clinical care and screening practices, but it may also have been influenced by follow-up bias, missing ophthalmic examinations, or changes in sample composition over time. For this reason, the temporal trend should be viewed as exploratory rather than as definitive evidence of true longitudinal change within the same individuals. Nevertheless, this favorable observed pattern may have been facilitated by earlier interventions and improved screening techniques [25].

This study has several strengths. It used a retrospective cohort design with an adequate sample size and prolonged follow-up, allowing reliable incidence estimation and long-term risk factor assessment. The use of BESTCare 2.0A, a comprehensive electronic medical record system, helped reduce missing data and supported systematic data collection. Furthermore, uniform grading of diabetic retinopathy based on the International Clinical Diabetic Retinopathy Severity Scale improved diagnostic consistency and study comparability [13]. The investigation of both cross-sectional and longitudinal relationships was further strengthened by the incorporation of multiple analytical techniques, including multivariable regression and Cox proportional hazards models, while the assessment of glycemic variability added depth beyond conventional glycemic measures.

From a public health perspective, these findings may help tertiary care facilities in Saudi Arabia develop more targeted DR screening strategies for patients with T1DM. Patients with longer diabetes duration, older age at diagnosis, hypertension, hyperlipidemia, or albuminuria may benefit from closer ophthalmologic surveillance and integrated multidisciplinary follow-up involving endocrinology, ophthalmology, nephrology, and primary care. Incorporating these risk factors into electronic medical record–based alerts may also support earlier referral and improve adherence to annual retinal screening. Future multicenter prospective studies are needed to validate these findings and guide national risk-based screening policies for diabetic retinopathy in Saudi Arabia.

However, several limitations should be considered. In addition to limiting causal inference, the retrospective design may introduce information and selection bias. Furthermore, the single-center tertiary care setting and consecutive non-probability sampling may limit the generalizability of our findings. Because King Abdulaziz Medical City serves National Guard Health Affairs beneficiaries and provides specialized ophthalmology care, the included population may differ from community-based or primary-care populations with T1DM in Saudi Arabia. In particular, patients with more complex systemic diseases or greater access to specialist ophthalmic evaluation may be over-represented. Moreover, certain factors, including smoking and family history, are prone to recall bias because they partially rely on self-report. Additionally, residual confounding from unmeasured variables, such as treatment adherence and socioeconomic status, cannot be ruled out. Although age-stratified analysis may provide further insight into DR risk patterns, subdividing the cohort into multiple age categories would reduce the number of participants and events within some subgroups, particularly for NVTDR and VTDR comparisons. Therefore, age-related effects were addressed through multivariable adjustment for age at T1DM diagnosis and diabetes duration. Future larger regional multicenter studies are needed to further evaluate age-stratified risk patterns among patients with T1DM. Lastly, the observed relationships may have been influenced by the use of averaged HbA1c readings and variations in follow-up time.

## 5. Conclusions

In conclusion, this study provides evidence on the incidence and risk factors of diabetic retinopathy in patients with type 1 diabetes mellitus within a tertiary-care cohort in Riyadh, Saudi Arabia. Older age at diagnosis, longer disease duration, hypertension, hyperlipidemia, and albuminuria were key variables associated with DR, highlighting the importance of cumulative metabolic and systemic vascular stress. The lack of an independent association between mean HbA1c and DR suggests that comorbidities and overall disease burden may have contributed more strongly to DR risk in this sample. These findings highlight the need for a multidisciplinary approach that emphasizes systemic risk factor management, glycemic control, and routine ophthalmic screening. Future prospective regional multicenter studies are needed to validate these findings.

## Figures and Tables

**Table 1:** Baseline Characteristics and Risk Factors by Diabetic Retinopathy Classification.

Variable	Control (*n* = 268)	Cases (*n* = 180) *		
		NVTDR (*n* = 52)	VTDR (*n* = 128)	*p*-Value
**Age (years)**, mean ± SD; median (IQR)	26.66 ± 12.10; 24.50 (19.00–30.25)	55.42 ± 17.92; 59.00 (40.25–69.25)	52.42 ± 16.40; 53.00 (39.75–64.00)	<0.0001
**BMI (kg/m^2^)**, mean ± SD; median (IQR)	25.01 ± 5.81; 24.55 (20.65–28.09)	32.13 ± 6.98; 31.93 (27.91–36.29)	30.25 ± 6.04; 29.41 (26.52–34.44)	<0.0001
**Age at T1DM diagnosis (years)**, mean ± SD; median (IQR)	11.23 ± 12.14; 8.00 (5.00–14.00)	41.04 ± 20.54; 42.00 (24.00–56.00)	36.65 ± 18.10; 37.00 (22.00–50.00)	<0.0001
**Duration of diabetes (years)**, mean ± SD; median (IQR)	16.17 ± 6.10; 14.75 (11.03–20.03)	16.31 ± 10.60; 11.92 (9.85–20.49)	16.71 ± 9.40; 14.48 (9.74–22.03)	0.0513
**Mean HbA1c (5 readings)**, mean ± SD; median (IQR)	9.61 ± 1.81; 9.42 (8.16–10.68)	9.29 ± 1.77; 8.87 (8.01–10.34)	9.56 ± 1.61; 9.54 (8.32–10.61)	0.4129
**HbA1c variability (SD)**, mean ± SD; median (IQR)	1.02 ± 0.77; 0.77 (0.51–1.22)	0.90 ± 0.74; 0.63 (0.45–1.09)	1.21 ± 0.89; 0.98 (0.55–1.49)	0.0189
**HbA1c variability (CV%)**, mean ± SD; median (IQR)	10.58 ± 7.84; 8.35 (5.52–12.70)	9.61 ± 7.53; 7.45 (5.02–11.18)	12.58 ± 9.33; 10.36 (6.36–15.57)	0.0246
**Total Cholesterol**, mean ± SD; median (IQR)	4.70 ± 1.01; 4.57 (4.04–5.16)	4.27 ± 1.09; 4.11 (3.46–4.88)	4.31 ± 1.16; 4.17 (3.67–4.92)	<0.0001
**HDL Cholesterol**, mean ± SD; median (IQR)	1.35 ± 0.32; 1.33 (1.14–1.54)	1.19 ± 0.27; 1.18 (0.98–1.39)	1.13 ± 0.31; 1.10 (0.94–1.25)	<0.0001
**Gender**, *n* (%)				0.1293
Male	120 (44.8%)	17 (32.7%)	63 (49.2%)	
Female	148 (55.2%)	35 (67.3%)	65 (50.8%)	
**BMI Categories**, *n* (%)				<0.0001
Underweight (<18.5)	28 (10.5%)	2 (3.8%)	3 (2.3%)	
Normal Weight (18.5–24.9)	111 (41.7%)	5 (9.6%)	19 (14.8%)	
Overweight (25.0–29.9)	81 (30.5%)	14 (26.9%)	44 (34.4%)	
Obesity (≥30.0)	46 (17.3%)	31 (59.6%)	62 (48.4%)	
**Family history of DM (any)**, *n* (%)	147 (54.9%)	38 (73.1%)	94 (73.4%)	0.0004
**Family history of DM (T1DM)**, *n* (%)	46 (17.2%)	7 (13.5%)	29 (22.7%)	0.2632
**Family history of DM (T2DM)**, *n* (%)	113 (42.2%)	31 (59.6%)	65 (50.8%)	0.0377
**Family history of DM (T1DM and T2DM)**, *n* (%)	12 (4.5%)	0 (0.0%)	0 (0.0%)	0.0159
**Hypertension**, *n* (%)	19 (7.1%)	34 (65.4%)	90 (70.3%)	<0.0001
**Hyperlipidemia**, *n* (%)	114 (42.5%)	42 (80.8%)	104 (81.2%)	<0.0001
**Albuminuria**, *n* (%)	33 (12.3%)	17 (32.7%)	58 (45.3%)	<0.0001
**Smoking**, *n* (%)	15 (5.6%)	6 (11.5%)	16 (12.5%)	0.0432

Values are presented as mean ± standard deviation, median (interquartile range), or number (%), as appropriate. * Table 1 includes 448 participants with complete available baseline data for the descriptive comparison. One patient in the case group had missing baseline covariate data and was therefore excluded from this table only; however, this patient was retained in analyses and figures for which the required data were available. Abbreviations: BMI, body mass index; CV, coefficient of variation; DM, diabetes mellitus; HbA1c, glycated hemoglobin; HDL, high-density lipoprotein; IQR, interquartile range; NVTDR, non-vision-threatening diabetic retinopathy; SD, standard deviation; T1DM, type 1 diabetes mellitus; T2DM, type 2 diabetes mellitus; VTDR, vision-threatening diabetic retinopathy.

**Table 2:** Incidence Rate of Diabetic Retinopathy.

Group	Incidence Rate (per 1000 Person-Years)	95% CI (Lower–Upper)
Overall	92.66	79.03–106.83
Female	91.75	73.59–110.86
Male	93.87	73.57–115.43

Incidence rate of diabetic retinopathy overall and by gender, expressed per 1000 person-years with 95% confidence intervals. Abbreviations: CI, confidence interval.

**Table 3:** Primary and Secondary Multivariable Logistic Regression Models for Diabetic Retinopathy and Vision-Threatening Diabetic Retinopathy.

Risk Factor	Adjusted OR for DR (95% CI) *	*p*-Value	Adjusted OR for VTDR (95% CI) **	*p*-Value
Age at T1DM diagnosis	1.08 (1.05–1.11)	<0.0001	0.98 (0.96–1.01)	0.1959
Duration of diabetes	1.08 (1.03–1.12)	0.0007	1.00 (0.96–1.04)	0.9144
Mean HbA1c	1.08 (0.90–1.29)	0.3953	1.01 (0.82–1.26)	0.8977
HbA1c variability	1.05 (0.75–1.48)	0.7674	1.46 (0.91–2.35)	0.1178
Hypertension	3.73 (1.70–8.15)	0.0010	1.83 (0.79–4.24)	0.1603
Hyperlipidemia	2.38 (1.25–4.54)	0.0081	1.15 (0.48–2.77)	0.7522
Albuminuria	3.79 (1.85–7.77)	0.0003	1.53 (0.75–3.11)	0.2441
Smoking	2.10 (0.79–5.64)	0.1388	0.85 (0.29–2.46)	0.7591
Obesity (BMI ≥ 30.0)	1.68 (0.85–3.32)	0.1362	0.68 (0.34–1.38)	0.2852

Results are presented as adjusted odds ratios (ORs) with 95% confidence intervals (CIs). * Model A (DR): *n* = 443; outcome variable: DR, comparing cases with controls. ** Model B (VTDR): *n* = 179; outcome variable: VTDR, comparing VTDR with non-vision-threatening diabetic retinopathy among DR cases. Abbreviations: BMI, body mass index; CI, confidence interval; DR, diabetic retinopathy; HbA1c, glycated hemoglobin; OR, odds ratio; T1DM, type 1 diabetes mellitus; VTDR, vision-threatening diabetic retinopathy.

**Table 4:** Complementary Cox Proportional Hazards Models for Time to Diabetic Retinopathy and Vision-Threatening Diabetic Retinopathy.

Risk Factor	HR for DR (95% CI)	*p*-Value	HR for VTDR (95% CI)	*p*-Value
Age at T1DM diagnosis	1.03 (1.01–1.04)	0.0001	1.02 (1.00–1.03)	0.0328
Mean HbA1c	1.06 (0.95–1.18)	0.3024	1.04 (0.91–1.18)	0.5555
HbA1c variability	0.85 (0.70–1.04)	0.1118	0.94 (0.75–1.17)	0.5707
Hypertension	1.36 (0.88–2.10)	0.1684	1.66 (1.00–2.75)	0.0485
Hyperlipidemia	0.68 (0.44–1.04)	0.0748	0.75 (0.44–1.26)	0.2723
Albuminuria	1.08 (0.78–1.51)	0.6400	1.24 (0.84–1.83)	0.2796
Smoking	1.17 (0.68–2.04)	0.5690	1.03 (0.53–1.99)	0.9257
Obesity (BMI ≥ 30.0)	1.02 (0.72–1.44)	0.9057	0.93 (0.62–1.40)	0.7413

Results are presented as hazard ratios with 95% confidence intervals. Abbreviations: CI, confidence interval; DR, diabetic retinopathy; HR, hazard ratio; T1DM, type 1 diabetes mellitus; VTDR, vision-threatening diabetic retinopathy.

**Table 5:** Exploratory Multivariable Logistic Regression Model for Proliferative Diabetic Retinopathy Versus Non-Proliferative Diabetic Retinopathy Among Diabetic Retinopathy Cases.

Risk Factor	Adjusted OR for PDR *	95% CI	*p*-Value
Age at T1DM diagnosis	0.96	0.94–0.99	0.006
Duration of diabetes (>10 vs. ≤10 years)	0.61	0.26–1.39	0.238
HbA1c (Poor vs. good)	0.84	0.22–3.29	0.806
HbA1c (moderate vs. good)	0.92	0.24–3.52	0.901
Hypertension (Yes vs. no)	1.38	0.55–3.44	0.494
Hyperlipidemia (Yes vs. no)	1.38	0.53–3.60	0.511
Albuminuria	1.86	0.86–4.04	0.116
Smoking	0.92	0.31–2.76	0.879
BMI (underweight/normal vs. ≥30.0)	1.94	0.66–5.71	0.230
BMI (overweight vs. ≥30.0)	0.85	0.36–2.02	0.707

Results are presented as adjusted odds ratios with 95% confidence intervals. * The model compares proliferative diabetic retinopathy versus non-proliferative diabetic retinopathy among patients with diabetic retinopathy. Non-proliferative diabetic retinopathy includes mild, moderate, and severe non-proliferative diabetic retinopathy. Glycated hemoglobin was categorized as good control (<7.5%), moderate control (7.5–10.0%), and poor control (>10.0%), with good control used as the reference category. Body mass index was categorized as underweight (<18.5 kg/m^2^), normal weight (18.5–24.9 kg/m^2^), overweight (25.0–29.9 kg/m^2^), and obesity (≥30.0 kg/m^2^), with obesity used as the reference category. For binary variables, odds ratios compare the presence versus absence of the condition. Abbreviations: BMI, body mass index; CI, confidence interval; HbA1c, glycated hemoglobin; OR, odds ratio; PDR, proliferative diabetic retinopathy; T1DM, type 1 diabetes mellitus.

**Table 6:** Distribution of Diabetic Retinopathy Stages Across Sequential Available Ophthalmic Assessments.

DR Stage	Assessment 1 (*n* = 176)	Assessment 2 (*n* = 156)	Assessment 3 (*n* = 125)
Mild NPDR	51 (29.0%)	33 (21.2%)	21 (16.8%)
Moderate NPDR	57 (32.4%)	52 (33.3%)	43 (34.4%)
Severe NPDR	25 (14.2%)	24 (15.4%)	19 (15.2%)
PDR	43 (24.4%)	47 (30.1%)	42 (33.6%)

Assessment 1, Assessment 2, and Assessment 3 represent the first, second, and third available documented ophthalmic assessments during follow-up, respectively, rather than predefined follow-up intervals. Because the number of patients assessed differed across assessment points, percentages were calculated using the total number of available staged assessments at each corresponding assessment point. Therefore, the table describes the observed distribution of diabetic retinopathy stages among available records and should not be interpreted as true longitudinal change within the same individuals. Abbreviations: NPDR, non-proliferative diabetic retinopathy; PDR, proliferative diabetic retinopathy.

## Data Availability

The data supporting the findings of this study are not publicly available due to institutional and privacy restrictions but are available from the corresponding author upon reasonable request.

## Abbreviations

The following abbreviations are used in this manuscript:

ACR	Albumin-to-creatinine ratio
ATP III	Adult Treatment Panel III
BMI	Body mass index
CI	Confidence interval
CV	Coefficient of variation
DM	Diabetes mellitus
DME	Diabetic macular edema
DR	Diabetic retinopathy
HbA1c	Glycated hemoglobin
HDL	High-density lipoprotein
HR	Hazard ratio
IQR	Interquartile range
KAIMRC	King Abdullah International Medical Research Center
KAMC	King Abdulaziz Medical City
LDL	Low-density lipoprotein
NGHA	National Guard Health Affairs
NPDR	Non-proliferative diabetic retinopathy
NVTDR	Non-vision-threatening diabetic retinopathy
OR	Odds ratio
PDR	Proliferative diabetic retinopathy
SD	Standard deviation
SPSS	Statistical Package for the Social Sciences
T1DM	Type 1 diabetes mellitus
T2DM	Type 2 diabetes mellitus
VTDR	Vision-threatening diabetic retinopathy

## Appendix A

**Table A1:** Categorized-Variable Multivariable Logistic Regression Models for Diabetic Retinopathy and Vision-Threatening Diabetic Retinopathy.

Risk Factor	Adjusted OR for DR *	95% CI	*p*-Value	Adjusted OR for VTDR **	95% CI	*p*-Value
Age at T1DM diagnosis	1.08	1.05–1.10	<0.001	0.98	0.96–1.01	0.124
Duration of diabetes (>10 vs. ≤10 years)	1.89	0.92–3.91	0.085	0.95	0.45–2.02	0.898
HbA1c (Poor vs. good)	2.41	0.78–7.51	0.128	1.31	0.40–4.25	0.652
HbA1c (moderate vs. good)	1.71	0.55–5.27	0.351	1.36	0.43–4.31	0.600
Hypertension (Yes vs. no)	5.01	2.34–10.73	<0.001	1.96	0.83–4.64	0.125
Hyperlipidemia (Yes vs. no)	2.44	1.30–4.60	0.006	1.22	0.50–2.97	0.654
Albuminuria	3.31	1.67–6.56	<0.001	1.66	0.82–3.38	0.161
Smoking	2.28	0.84–6.19	0.105	0.92	0.32–2.64	0.873
BMI (underweight/normal vs. ≥30.0)	0.58	0.26–1.27	0.170	1.60	0.53–4.77	0.402
BMI (overweight vs. ≥30.0)	0.55	0.27–1.15	0.111	1.40	0.65–3.02	0.392

Results are presented as adjusted odds ratios with 95% confidence intervals. * The diabetic retinopathy model compares cases versus controls. ** The vision-threatening diabetic retinopathy model compares vision-threatening diabetic retinopathy versus non-vision-threatening diabetic retinopathy among diabetic retinopathy cases. Glycated hemoglobin was categorized as good control (<7.5%), moderate control (7.5–10.0%), and poor control (>10.0%), with good control used as the reference category. Body mass index was categorized as underweight (<18.5 kg/m^2^), normal weight (18.5–24.9 kg/m^2^), overweight (25.0–29.9 kg/m^2^), and obesity (≥30.0 kg/m^2^), with obesity used as the reference category. For binary variables, absence of the condition was used as the reference category. Abbreviations: BMI, body mass index; CI, confidence interval; DR, diabetic retinopathy; HbA1c, glycated hemoglobin; OR, odds ratio; T1DM, type 1 diabetes mellitus; VTDR, vision-threatening diabetic retinopathy.
